# A Streamlined, Automated Protocol for the Production of Milligram Quantities of Untagged Recombinant Rat Lactate Dehydrogenase A Using ÄKTAxpress^TM^

**DOI:** 10.1371/journal.pone.0146164

**Published:** 2015-12-30

**Authors:** Matthew W. Nowicki, Elizabeth A. Blackburn, Iain W. McNae, Martin A. Wear

**Affiliations:** The Edinburgh Protein Production Facility (EPPF), Wellcome Trust Centre for Cell Biology (WTCCB), University of Edinburgh, King's Buildings, Max Born Crescent, Mayfield Road, Edinburgh, United Kingdom; National Center for Toxicological Research, US Food and Drug Administration, UNITED STATES

## Abstract

We developed an efficient, automated 2-step purification protocol for the production of milligram quantities of untagged recombinant rat lactate dehydrogenase A (rLDHA) from *E*. *coli*, using the ÄKTAxpress™ chromatography system. Cation exchange followed by size exclusion results in average final purity in excess of 93% and yields ~ 14 milligrams per 50 ml of original cell culture in EnPresso B media, in under 8 hrs, including all primary sample processing and column equilibration steps. The protein is highly active and coherent biophysically and a viable alternative to the more problematic human homolog for structural and ligand-binding studies; an apo structure of untagged rLDHA was solved to a resolution 2.29 Å (PDB ID 5ES3). Our automated methodology uses generic commercially available pre-packed columns and simple buffers, and represents a robust standard method for the production of milligram amounts of untagged rLDHA, facilitating a novel fragment screening approach for new inhibitors.

## Introduction

As the breadth and pace of large biomedical, drug discovery and structural genomics programs has increased, the requirement for protein production strategies to be reliable and robust has become more and more critical. The need to regularly generate milligrams of highly pure, biophysical coherent and active protein, with little batch variation has resulted in modern chromatography instruments becoming increasingly automated [1–4], separation media and expression/production technologies becoming increasingly sophisticated and routinely implemented outside of specialist protein production labs, and effective preparative protein purification becoming more routine [5–12]. Most methodologies tend to be protein/protein-family specific, utilising specific differences in biophysical properties particular to the individual protein(s), making them hard to translate efficiently into generic or high-throughput purification strategies. Coupled to this is the fact that the majority of purification strategies are multistep [13–18]) and most lab-scale chromatography instrumentation normally handles only a single step at a time, and thus can involve significant user intervention and time-consuming manual processing. High-end conventional LC systems can be adapted to run automated multi-step protocols, either by adaptive software/hardware additions [19] or by intelligent peak collection and sample diversion/loading strategies using the existing hardware [20]. However, this automation/parallelization is less than straightforward for non-experts to “standardise”. There are often significant operational differences between how different labs use them (delay volumes, tubing diameters, valve configurations, column types etc.) that make this adaptive automated method development harder to routinely implement.

The ÄKTAxpress™ liquid chromatography system (GE Healthcare) was the first commercially available standardised lab-scale system designed specifically to run automated, parallelized, multi-step (up-to 4) purification protocols [3, 21, 22]. The non-modular design, with identical flow path delay volumes, gradient delay volumes, pre-defined commercially standardised column types and the same sets of limited user-editable protocol design parameters, means in theory, that there is no appreciable difference in way the instruments are run between laboratories. The vast bulk of ÄKTAxpress™ protocols utilize affinity chromatography as the first step [3, 23], as enrichment best fits the sample handling restrictions for subsequent protocol steps. However, affinity tags, and optimizing their subsequent removal (regularly pertinent for structural analysis), occasionally causes as many problems as the development of the purification protocol itself (this is even the case for the simple poly-histidine tag [21, 22, 24]). To our knowledge, only a handful of generic protocols have been published for the purification of untagged proteins using such automated systems [21].

One protein of particular interest to us as a potential therapeutic anti cancer target is mammalian lactate dehydrogenase A (LDHA). For decades, it has been known that certain tumors alter the metabolism of the transformed cells they are in; the so-called Warburg effect [25, 26]. Many tumor-transformed tissues have severely altered glucose metabolism and greatly increase the rate of glucose uptake, relative to normal cells, and metabolise this via glycolysis and not by mitochondrial oxidative phosphorylation [27–36]. In contrast to normal tissues, such glycolytic glucose depletion occurs in cancer cells even in abundant oxygen levels [27]. Warburg’s “aerobic glycolysis” [25, 26] is an attractive model marker that distinguishes between tumors and healthy tissues, and one potentially exploitable for development of new anti-cancer agents [28, 37–43]. LDHA (also known as LDH-M and LDH-5) is a homotetrameric enzyme that catalyzes the cytosolic conversion of pyruvate to lactate in the final step of glycolysis, oxidizing nicotinamide adenine dinucleotide (NADH) in the process [37]. Elevated LDHA levels are integrally associated with many cancer types and it appears likely to be a principal factor in the altered metabolisms required for the growth and proliferation of certain tumors [27–29, 37, 39, 44, 45]. These observations have highlighted LDHA as an attractive target for new anti-cancer agents for use against glycolytic tumors [38, 39].

We found that the human LDHA protein was more problematic to work with than the rat homologue (despite structures being available, PDB code 1i10 [46]), similar to the observations of other labs [38, 39]. In our hands the presence of his-tags, especially at the N-terminus, adversely affected the levels of soluble expression, protein activity, tag-cleavage and hampered the subsequent ability to generate a robust protein crystal structure rationale for our small molecule/fragment inhibitor studies. It is unclear why this should be the case, but on average the levels of soluble expression and activity of the tagged protein were ~ 15–18% less than the untagged. Partial removal of the tag–at best 50% efficient–is very likely explained by occlusion of the N-terminus and protease site in quaternary structure of the LDHA. This material did not yield crystals.” Considering extremely high sequence identity between the human and rat LDHA sequences (94% identity), the fact that the cofactor and substrate binding sites are identical, and recent structural work from the literature with the rat homologue [38, 39], we developed a very efficient, automated purification protocol for the production of milligram quantities of very pure, untagged rat LDHA (rLDHA), as an alternative to the human protein, from *E*. *coli*, using the ÄKTAxpress™ chromatography system. The 2-step protocol (cation exchange and size exclusion) results in a typical final purity of ≥ 93% and final yield of ~ 14 mg per 50 ml of original cell culture in EnPresso B media, in under 8 hrs. The protein is highly active and biophysically coherent, and allowed us to develop a novel rationale for generation of a soakable protein crystal system (a structure of rLDHA was solved to 2.29 Å resolution; PDB ID 5ES3). The automated protocol uses standard commercially available pre-packed columns and simple buffers and represents a robust generic method for the automated production of untagged rLDHA.

## Materials and Methods

### Materials

All chemicals used were of the highest grade available commercially.

### Plasmid construction

The codon optimsed (Geneart) ORF corresponding to amino acids 1–332 of full-length wild type rat lactate dehydrogenase A (rLDHA) was first cloned into pDONR-221 (Invitrogen) and subsequently sub-cloned into pDEST-14 (Invitrogen) using standard GATEWAY methodology and the final vector sequence verified.

### Protein Expression and Purification

Over-expression of rLDHA was achieved by addition of IPTG to 1 mM to OverExpress C41 BL21(DE3) *E*. *coli* (Lucigen), transformed with the rLDHA expression plasmid, grown shaking (260 rpm) at 30°C for 24 hrs in 50 ml of EnPresso B media (BioSilta™) in a 500 ml flask. In our laboratory this strain of *E*. *coli* (a phenotypic mutant selected for conferring tolerance to toxic proteins) consistently performs better, in terms of soluble expression, than standard BL21(DE3) strains with both non-toxic and toxic proteins. Cells were harvested by centrifugation at 4,000 x g for 10 min at 4°C and cell pellets used immediately or flash frozen in liquid nitrogen and stored at -70°C.

**Fig 1:**
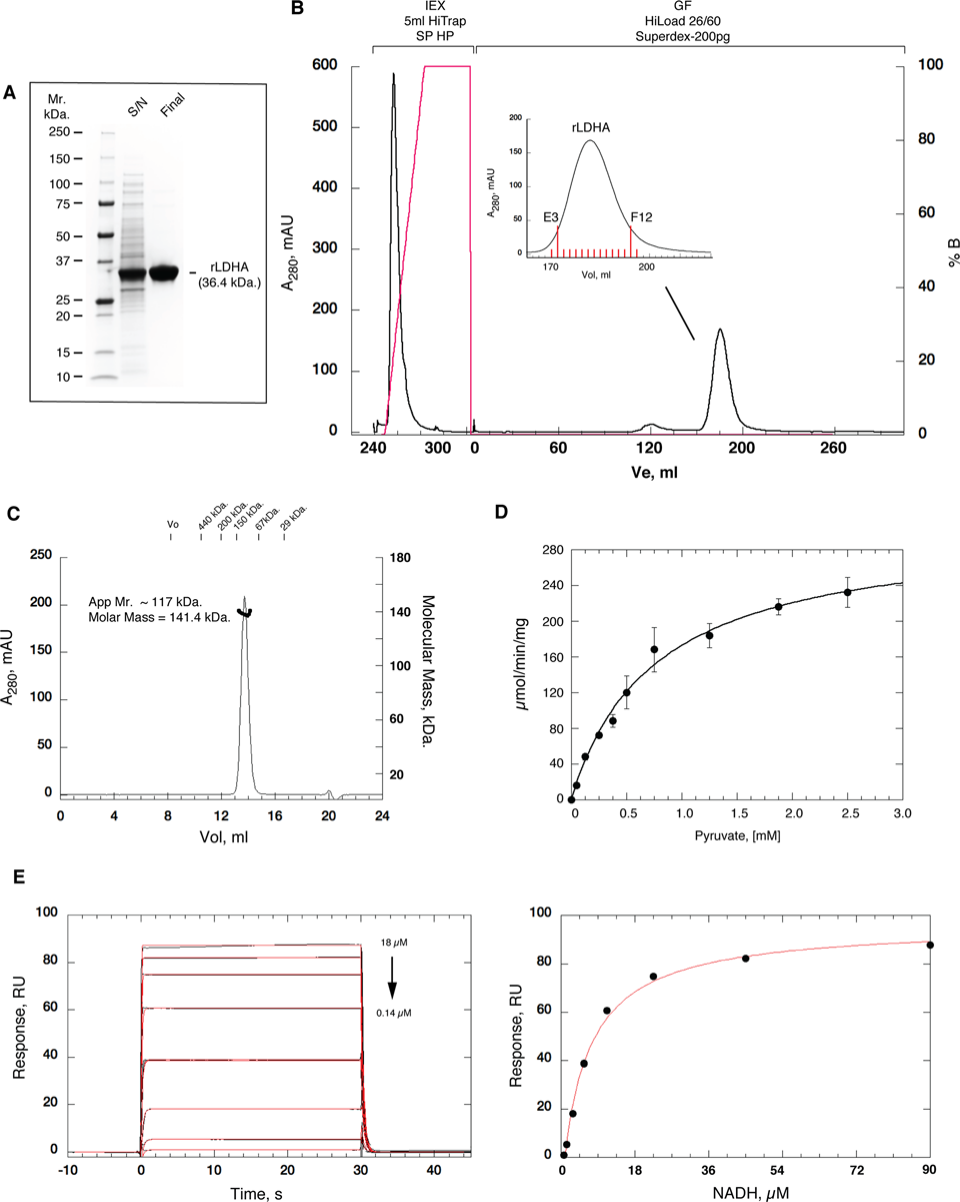
Automated Purification of untagged rLDHA. (A) SDS polyacrylamide gel (4–20% gradient) illustrating the typical level of soluble rLDHA extracted in the clarified lysate and final purified sample, from OverExpress C41 BL21(DE3) *E*. *coli*. S/N, clarified supernatant; Final; final pooled sample. 5 μg total load in each lane. (B) Typical chromatogram for the automated 2-step purification of rLDH using ÄKTAXpress™. The pre-packed columns used are illustrated above the corresponding section of the chromatogram; IEX—ion-exchange, GF—gel filtration. Solid black; A_280nm_ in mAU (left axis). Solid red; NaCl gradient in % *Buffer-B* (right axis). The inset details the region of the gel filtration column elution from which fractions were collected. Indicated fractions E3 –F12 were pooled. (C) Mono-dispersity and size analysis. Size-exclusion chromatography multi-angled light scattering (SEC MALS) of rLDH protein. Size-exclusion chromatography (ÅKTA-Micro; GE Healthcare) coupled to UV, static light scattering and refractive index detection (Viscotec SEC-MALS 20 and Viscotek RI Detector:VE3580; Malvern Instruments) were used to determine the molecular mass of rLDH in solution. 100 μL of 1 mg.mL^-1^ rLDH was run on a Superdex-200 10/300 GL (GE Healthcare) size exclusion column pre-equilibrated in *Buffer-C*, at 22°C with a flow rate of 0.5 mL.min^-1^. Light scattering, refractive index (RI) and A_280nm_ were analysed by a homo-polymer model (OmniSEC software, v 5.1; Malvern Instruments) using the following parameters for rLDH protein: ∂A / ∂c at 280nm 1.19 AU.mL.mg^-1^ and ∂n / ∂c of 0.185 mL.g^-1^. rLDH protein elutes a single sharp peak with apparent molecular mass of ~ 117 ± 15 kDa. and an Rs of 4.9 ± 0.2 nm (mean ± SD, n = 3). Elution position for standards are shown above the chromatograph. The molecular mass average across the elution profile is 141.4 kDa. with excellent mono-dispersity (Mw/Mn = 1.001). The theoretical molecular weight of tetrameric rLDH is 145.6 kDa. (monomer = 36.4 kDa.) Protein concentration was determined by measurement of absorbance at 280 nm and calculated using the extinction coefficient of 43,600 M^-1^.cm^-1^. (D) Specific activity of rLHDA verified by oxidation of NADH as function of pyruvate concentration. Assay performed at 30°C in *Buffer-C* supplemented with 3 mM NADH monitoring at 340 nm with 0.01 μg of rLDHA. *K*_m_ value (fit to Eq 1; solid line) is 0.53 ± 0.08 mM for pyruvate and specific activity is 310 μmol.mg^-1^.min^-1^. (E) Characterisation of the interaction of NADH with rLDHA using BIAcore T200. Active rLDHA surfaces were generated by capture of biotinylated protein on SA sensor surfaces (GE Healthcare). Between 3,900 RU– 7800 RU were immobilized on three separate surfaces on a SA sensor chip. Left Panel. Representative reference corrected SPR binding curves (black), monitored on a surface of 5,272 RU of rLDHA for various dilution series (in the 90 μM– 0.7 μM range) of NADH at 25°C in *Buffer-C*, supplemented with 0.05% surfactant P20. The apparent on- and off-rate constants (above) were determined by globally fitting (red) a 1:1 kinetic binding model, with mass transport considerations, to three different density surfaces (3,122 RU, 5,272 RU & 7810 RU) simultaneously, using the analysis software (v1.02, GE Healthcare) supplied with the instrument. The 1.125 μM sensorgram shows two replicate runs. Mean values (n = 3, ± SD) determined for the on-rate (*k*_+_), off-rate (*k*_-_) and equilibrium dissociation constants (*K*_d_) are 0.38 ± 0.06 μM^-1^.s^-1^, 2.7 ± 0.6 s^-1^ and 7 ± 0.6 μM, respectively. Right Panel. Steady-state affinity determination for NADH binding to rLDHA, fit using a 1:1 Langmuir interaction model (solid red line), gives a mean *K*_d_ value of 6.4 ± 0.3 μM.

All purification was performed on ÄKTAxpress™ (GE Healthcare) equipment at 6°C. The ÄKTAxpress™ instruments were used in a standard cold-run configuration and 10 ml collection loops, with modifications described below. The cell pellet from 50 ml of EnPresso B culture was re-suspended in *Buffer-A* (100 mM NaOAc, pH 5.0; 50 mM NaCl) to 10% weight per volume, supplemented with 1 protease inhibitor tablet (cOmplete™, EDTA-free, Roche). Lysis was performed at 6°C by a single passage through a Constant Systems Cell Disruptor TS Series Benchtop instrument (Constant Systems) set to 25 kPsi, and cellular debris removed by centrifugation at 50,000 × g for 45 min at 4°C. Following lysis, the clarified supernatant was applied to an ÄKTAXpress™ system fitted with 5 ml HiTrap SP HP ion-exchange (IEX) and HiLoad 26/60 Superdex-200pg size exclusion (SEC) columns (GE Healthcare), attached to the system with the default system lengths of 1.0 mm i.d. Tefzel® tubing. Flow rates were 5 ml.min^-1^ and 3.2 ml.min^-1^, IEX and SEC steps, respectively. Proteins were detected by absorbance at 280 nm. Following sample loading, unbound material was washed through the IEX matrix with a further 25 column volumes of *Buffer-A*. Elution from the IEX matrix was performed by a 10 column volume linear gradient from 0–100% *Buffer-B* (100 mM NaOAc, pH 5.0; 1 M NaCl), with default peak collection parameters for level and slope, collecting 10 ml into a single loop; rLDHA invariably eluted between 18% - 46% *Buffer-B*. The contents of the IEX loop were loaded onto the SEC column with a *peak-injection-flush-volume* of 13 ml, and eluted in *Buffer-C* (100 mM HEPES pH 7.5; 150 mM NaCl), with peak collection set to start after 0.26 column volumes, collecting 2 ml fractions throughout with peak collection default parameters for level and slope. Invariably, fractions E3 –F12 of the rLDHA peak were pooled, concentrated to ~ 1 mg.ml^-1^ and stored at 4°C on ice. rLDHA was routinely in excess of 93% pure as judged by densitometric analysis of SDS-polyacrylamide gels (Fig 1A) and verified as full length protein by mass spectrometry. An average of 14 mg final yield was routinely obtained from 50 ml of EnPresso B culture media.

### Mono-dispersity and size analysis

Size-exclusion chromatography multi-angled light scattering (SEC-MALS) of rLDH protein. Size-exclusion chromatography (ÄKTA-Micro; GE Healthcare) coupled to UV, static light scattering and refractive index detection (Viscotec SEC-MALS 20 and Viscotek RI Detector VE3580; Malvern Instruments) were used to determine the molecular mass of rLDH in solution. Multiple injections of 100 μL of 1 mg.ml^-1^ rLDHA were run on a Superdex-200 10/300 GL (GE Healthcare) size exclusion column pre-equilibrated in *Buffer-C* at 22°C with a flow rate of 0.5 ml.min^-1^. Light scattering, refractive index (RI) and A_280nm_ were analysed by a homo-polymer model (OmniSEC software, v5.02; Malvern Instruments) using the following parameters for rLDH protein: ∂A / ∂c at 280nm 1.19 AU.ml.mg^-1^, ∂n / ∂c of 0.185 ml.g^-1^ and buffer RI value of 1.334. Mass distribution analysis of rLDHA protein sample by dynamic light scattering (DLS) (data not shown) was performed on a Zetasizer APS (Malvern) with 5 repeat runs of 60 μl (0.5 mg.ml^-1^) in *Buffer-C* at 25°C, with a 120 s equilibration.

### rLDHA enzymatic assay

Reaction velocity was determined by measuring the decrease in the absorbance at 340 nm resulting from the oxidation of NADH [47]. 1 unit of enzymatic activity is defined as the reduction of 1 μmol of pyruvate per mg per minute, pH 7.5 at 30°C. Reactions were performed with 0.008–0.02 μg of rLDHA at 30°C in *Buffer-C* supplemented with 300 μM NADH in a total volume of 3 ml on a Jasco V-550 spectrophotometer. **Δ**A_340_/min values were converted to μmol.min^-1^.mg^-1^ using the extinction coefficient of 6,220 M^-1^.cm^-1^ for NADH. The initial reaction rates, V_o_ (in μmol.min^-1^.mg^-1^), were plotted against the concentration of pyruvate and the data least squares fit to Eq 1 using Kaleidagraph v4.1.3 software (Synergy Software, reading, PA);

$$V_{o}=\left(k_{cat}\times \left[rLDHA\right]\right)\times {\left[Pyruvate\right]}_{o}÷\left({\left[Pyruvate\right]}_{o}/K_{m}\right)$$ (1)

where [rLDHA] is the concentration of rLDHA, [Pyruvate]_o_ is the initial concentration of pyruvate, *k*_cat_ is the turnover number and *K*_m_ is the Michealis constant.

### Surface plasmon resonance (SPR)

SPR measurements were performed on a BIAcore T200 instrument (GE Healthcare). SA sensor chips were purchased from GE Healthcare. rLDHA was biotinylated using EZ-Link™ Sulfo-NHS-Biotin (Thermo Scientific) in *Buffer-C*, supplemented with 0.05% surfactant P20. Biotinylated rLDHA was then captured to the desired levels of by varying contact time with the sensor surface, at 30 μl.min^-1^ in *Buffer-C*, supplemented with 0.05% surfactant P20. SPR binding experiments with NADH were performed, in triplicate, at 25°C. A serial dilution concentration series of NADH ranging from 90 μM– 0.14 μM in *Buffer-C*, supplemented with 0.05% surfactant P20, was injected over the sensor surface, at 30 μl.min^-1^ with 30 s association and dissociation times. The apparent on- and off-rate constants (*k*_+_, *k*_-_) and the equilibrium dissociation constant (*K*_d_) were determined by globally fitting a 1:1 kinetic binding model, with mass transport considerations, to three different density surfaces (3,122 RU, 5,272 RU & 7810 RU) simultaneously, using the analysis software (v1.02, GE Healthcare) supplied with the instrument.

### Crystallisation and X-ray structure determination

LDH was concentrated to 22 mg.mL^-1^ and crystallised using the Morpheus® HT-96 crystallisation screen. Sitting drops were set up using the Douglas Instruments Oryx8 robot to pipette on to MRC 2 well crystallization plates (Swissci). The protein was mixed with the well solution (50 μL) in a 1:1 ratio (1 μL drop volume) and incubated at 18°C after plate sealing. A single crystal appeared in condition G10 (0.1 M Tris/Bicine pH 8.5, 20% ethylene glycol, 10% PEG 8000, 0.02 M of each of sodium formate, ammonium acetate, tri-sodium citrate, sodium/potassium tartrate and sodium oxamate) after a week. Data were collected at Diamond Light Source, UK, from a single crystal on beamline IO4 at 100 K and were processed using *XIA2* [48] utilizing XDS [49] The structure was solved by molecular replacement using *Phaser* [50] in the *PHENIX* suite [51] utilising a single chain from PDB ID 4AJE as the search model. Model building and initial refinment was carried out using the AutoBuild command in *PHENIX*, and and missing loops were fitted using the Fit Loop command. The structure was subjected to several rounds of refinement in *PHENIX* and manual refinement in *Coot* [52], including ligand fitting and loop re-modelling. Protein structure has the PDB ID 5ES3.

### Miscellaneous

SDS-PAGE was performed essentially as described [53]. The molecular mass of rLDHA is 36,400 Da. Protein concentration was determined by measurement of absorbance at 280 nm and calculated using the extinction coefficient 43,600 M^-1^.cm^-1^.

## Results and Discussion

We established a streamlined expression and automated 2-step methodology for the production of 10s of mg levels of very pure (typically in excess of 93%), highly biophysically coherent and active mammalian lactate dehydrogenase A (rat LDHA) from *E*.*coli*. (Fig 1). From extensive screens, we generated a robust and easily scalable set of conditions for high-level soluble expression of rLDHA for lab-scale production. Full-length codon optimised rLDHA in pDEST-14, transformed into OverExpress C41 BL21(DE3) *E*. *coli* and cultured in 50 mls of EnPresso B media, shaking at 260 rpm in 500 ml Erlenmeyer flasks, with 24 hr induction at 30°C by addition of IPTG to 1 mM, typically yields in excess of 20 mg per litre equivalent in the soluble extract (Table 1; Fig 1A). Culture in 50 ml EnPresso B media produces very high-density growth and results in biomass generation equivalent to that from 1 litre of traditional LB media grown under similar conditions. This reduced volume results in considerably easier and less time-consuming down-stream processing, in terms of centrifugation of the culture, lysis and clarification and the subsequent sample manipulation for chromatography. A further advantage of these optimized expression conditions is that scale-up production is substantially easier to implement than for traditional liquid media. Biomass production from 10 x 100 ml EnPresso B media cultures (in 1 litre Erlenmeyer shaker flasks grown under the above conditions) is equivalent to 10 litres of fermentation in LB media (300°C for 24 hrs). However, on average the soluble protein yield is 3 times greater, using our optimized conditions, than that obtained from 10 L of fermentation in LB media.

**Table 1:** Automated purification of rLDHA.

Fraction	Total protein (mg)^a^	Purity (%)^b^
**Soluble clarified lysate supernatant**	248	~ 13
**Pooled HiLoad Superdex200pg 26/60 fractions.**	14	≥ 93

Fractionation was performed on cell pellets obtained from *E*. *coli* cultured in 50 ml of EnPresso B media, which generates the equivalent biomass of 1 L of traditional liquid LB media.

^**a**^ Mean values from at least 2 individual repeat runs. Protein concentration in the supernatant and the pooled fractions was determined by A280 measurements.

^**b**^ average % purity determined by densitometry of appropriate lanes on reducing SDS-polyacrylamide gels (4–20% gradient, see Fig 1A).

We optimised a purification rationale to allow use of standard commercially available pre-packed columns and simple buffers (See Table 1) and translated it onto ÄKTAXpress™ system (GE Healthcare) fitted with 5 ml HiTrap SP HP IEX and HiLoad 26/60 Superdex-200pg SEC columns (GE Healthcare). Lysis and binding in *Buffer-A* (100 mM NaOAc, pH 5.0; 50 mM NaCl) allowed effective adsorption on the SP matrix, efficient removal and partitioning of essentially all of the contaminants and unbound material, and facilitated early elution of rLHDA during the gradient. Elution from the IEX matrix was performed by a 10 column volume linear gradient from 0–100% *Buffer-B* (100 mM NaOAc, pH 5.0; 1 mM NaCl), with default peak collection parameters for level and slope, collecting 10 ml into a single loop. rLDHA invariably eluted between 18% - 46% *Buffer-B* (Fig 1B). We found the minor loss of material arising from using the entire loop volume was more than compensated by the increased yield that resulted from collecting 10 ml of sample through the core of the eluted protein peak for the IEX column (similar to other work from our lab [21]). The contents of the IEX loop were loaded onto the SEC column with a *peak-injection-flush-volume* of 13 ml, and eluted in *Buffer-C* (100 mM HEPES pH 7.5; 150 mM NaCl), with peak collection set to start after 0.26 column volumes, collecting 2 ml fractions throughout with peak collection default parameters for level and slope. The GF elution profile shows a single major peak, eluting at 186 ± 2 ml, with good peak symmetry. Invariably, fractions E3 –F12 of the rLDHA peak were pooled (Fig 1B, inset), concentrated to ~ 1 mg.ml^-1^ and stored at 4°C on ice. rLDHA was verified as full length protein by mass spec. The methodology is very reproducible and results in a typical final purity of ≥ 93% and final yield of ~ 14 mg from 50 ml of original cell culture in EnPresso B media, in under 8 hrs (Table 1), with minimal manual processing.

Given the relative purity and amount of the material eluting from the IEX column, a 5 ml resin bed is apt to be able to cope with 4–5 times the initial starting material before dynamic breakthrough of rLDHA. This would potentially give final yields of 50–60 mgs of rLDHA protein, but without refinement of the long-term storage conditions, production of this amount of protein should be cautioned. Storage on ice at 4°C gave little evidence of loss of activity, aggregation or degradation in general for at least 3–4 weeks. After this, a slow loss of activity was observed. We found that rLDHA to be cold labile at lower temperatures and the protein did not cope particularly well with freezing (at either -20°C or -80°C) even in the presence of cyro-protectant (up to 20% glycerol and/or 20% PEG 3000) and at various concentrations ranging from 0.5–5 mg.ml^-1^. Variable loss of material and activity (20–70% for both) resulted from such conditions. Storage as an ammonium sulfate suspension (*Buffer-C* supplemented with (NH_4_)_2_SO_4_ to 3.2 M) works well for at least 8 weeks, but there are issues with reliable recovery of equal amounts of protein from the precipitate, even though specific activity and yield recovery were good; in excess of 85% typically. Nevertheless, the production and purification protocol we have developed allows for the rapid and reproducible production of 10s of milligrams of highly pure rLDHA and somewhat alleviates the long-term storage issues.

Before embarking on a series of crystallisation trials, we next extensively assessed the biophysical coherency and specific activity of the protein product purified by this method. Size-exclusion chromatography multi-angled light scattering (SEC-MALS) analysis was used to determine the molecular mass of rLDHA in solution. On a Superdex-200 10/300 GL (GE Healthcare) size exclusion column, rLDHA protein elutes a single sharp peak with apparent molecular mass of ~ 117 ± 15 kDa. and an R_s_ of 4.9 ± 0.2 nm (mean ± SD, n = 3). The molecular mass average across the elution profile is 141.4 ± 3.7 kDa. with excellent mono-dispersity (Mw/Mn = 1.001) (Fig 1C). This is in excellent agreement with theoretical molecular weight of 145.6 kDa. for tetrameric rLDHA (monomer = 36.4 kDa.). Mass distribution analysis was also carried out using dynamic light scattering (DLS) (data not shown). This further illustrated the very high mono-dispersity for the purified protein. Mean modal hydrodynamic radius (R_h_) was 4.3 ± 0.17 nm, with correlative molecular mass of 123 kDa. Hydropro [54] calculations using the 4AJ1.pdb crystal structure of tetrameric rat LDHA gave an R_h_ of 4.2 nm with an R_hmax_ of 4.98 nm. All of these data illustrate the very high quality of the purified protein and the robustness of the methodology for the production of very pure, mono-disperse, tetrameric rLDHA.

The purified protein also shows high specific activity. Typical results from enzyme assays following the oxidation of NADH as function of pyruvate concentration at 30°C are shown in Fig 1D. Mean *K*_m_ values for pyruvate were 0.53 ± 0.08 mM the mean specific activity was 310 ± 30 units.mg^-1^.min^-1^. These values are in good agreement with the literature for mammalian LDHA isoforms with *K*_m_ values for pyruvate which ranging from 0.05 to 1 mM and specific activity ranging from 50–600 units.mg^-1^.min^-1^ [39, 47]. The interaction of the co-factor NADH was also analyzed using SPR on a BIAcore T200 (Fig 1E). Specific activity of immobilized biotinylated rLDHA was again very high; typically in excess of 85%. Representative reference corrected SPR binding curves (black), for NADH binding to immobilised rLHDA are shown in Fig 1E (left panel). The mean apparent on-rate (*k*_+_), off-rate (*k*_-_) and equilibrium dissociation constants (*K*_d_), determined by globally fitting (red) a 1:1 kinetic binding model, with mass transport considerations, were 0.38 ± 0.06 μM^-1^.s^-1^, 2.7 ± 0.6 s^-1^ and 7 ± 0.6 μM, respectively. Steady-state affinity determination for NADH binding to rLDHA, gives a mean *K*_d_ value of 6.4 ± 0.3 μM. All of these kinetic and steady state values are in excellent agreement with the literature [39, 47].

**Fig 2:**
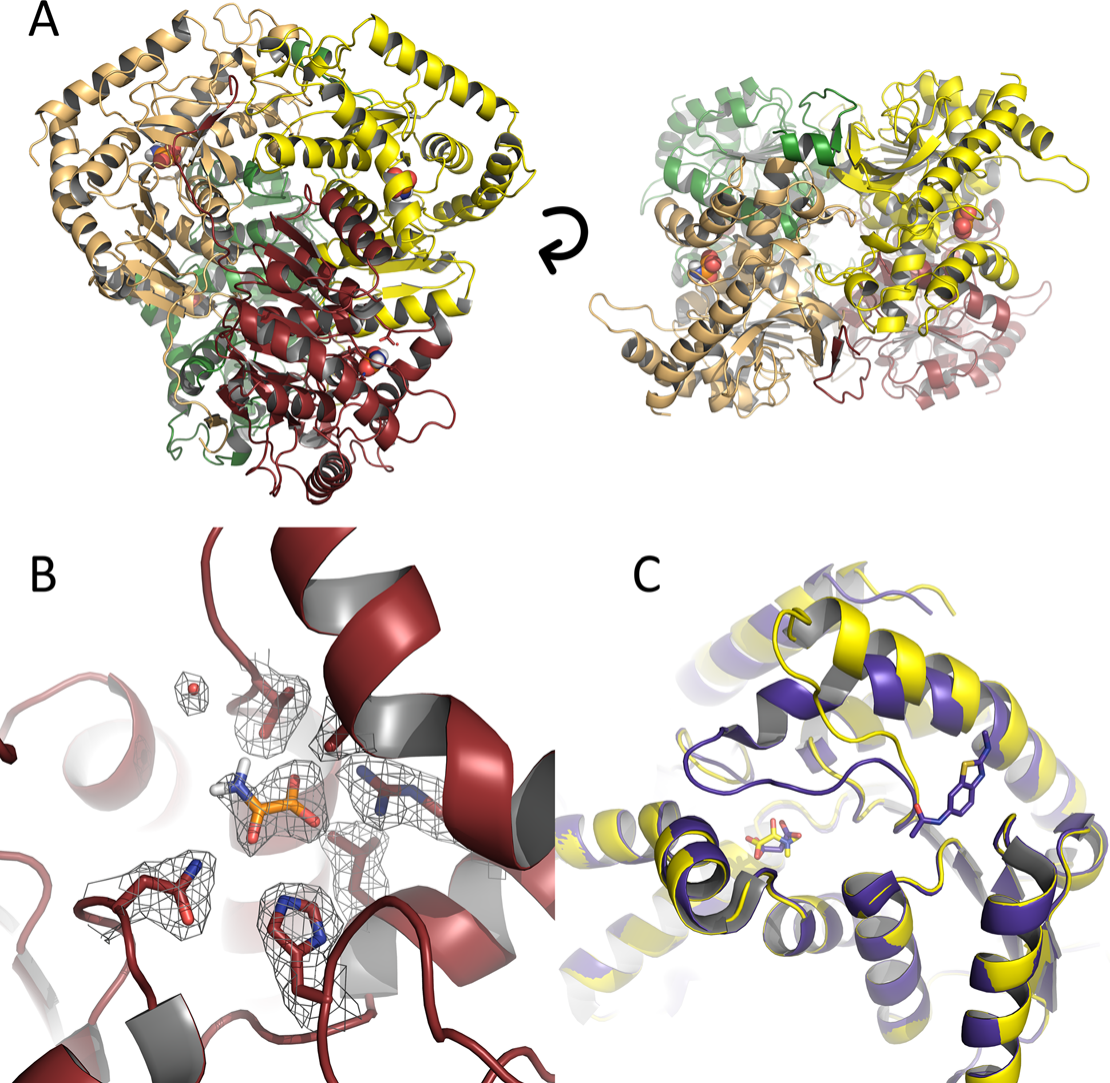
Crystal structure of rLDH complexed with oxamate. (A) Tetrameric biological unit as shown from a side view (left-hand panel) and a top view (right-hand panel) (PDB ID 5ES3). (B) Lactate binding site of rLDH with oxamate bound. Density contoured at 1σ. (C) Single subunit overlay of oxamate-bound open rLDH structure (yellow) and closed structure of 4AJ1with malonate and inhibitor bound.

We next embarked on development of a robust protein crystallisation rationale for use in soaking and co-crystallisation of fragments and small molecules for development of inhibitors for LDHA. Crystals grew using the commercially available Morpheus® screen with diffracting crystals appearing where oxamate (a known inhibitor of LDH [55] was present as an additive. The structure (PDB ID 5ES3) was solved to a resolution of 2.29 Å (Table 2) with the asymmetric unit containing 2 complete tetramers (Fig 2A). Upon close inspection both tetramers appeared in a ‘fully open’ conformation (all subunits in the tetramer adopting an open conformation with respect to the active site loop) despite the presence of electron density in the lactate binding site. The electron density was attributed to the presence of oxamate in the crystallization conditions. Oxamate was therefore modelled in to the density present in each of the 8 subunits in the assymetric unit (Fig 2B). Of note, to our knowledge this is the first example of an LDH crystal structure where oxamate (or lactate or oxalate) is bound without the presence of a co-factor (NAD/NADH) or co-factor mimic. Moreover, with the exception PDBID 1A5Z (*Thermotoga maritima* LDH [56]) our structure is also the first to have oxamate bound yet still be in a ‘fully open’ conformation. This conformation is depicted in Fig 2C where chain A from our structure is overlaid with another Rat LDH (PDBID 4AJ1) where malonate is bound in the presence of an NAD-mimic [38]. In the *T*. *maritima* structure, the open conformation is caused by a Cd^2+^ ion binding in the active site and stopping the loop from closing. These conformational state changes are important to charcaterise as they present very different molecular contacts surfaces for the binding of small molecules and fragments.

**Table 2:** Crystallographic data.

	LDH:oxamate
*data collection*	
resolution (Å)	2.29
space group	*P*2_1_2_1_2_1_
unit cell (Å)	
*a*	84.1
*b*	146.6
*c*	284.9
unique reflections^*a*^	158361 (11483)
completeness (%)^*a*^	100 (99)
R_merge_ (%)^*a*^	12.7 (70.2)
I/σ^*a*^	11.9 (2.7)
*refinement*	
*R* (%)	17.0
*R*_free_ (%)	20.3
all atoms used in refinement	21652
water	1321
*Validation* ^*b*^ (% of all residues)	
favoured	98.0
allowed	100.0
disallowed	0.0

^*a*^Values in parentheses refer to the highest-resolution shell.

^*b*^Validation was performed using MolProbity. PDB ID 5ES3

We have developed reliable and robust automated protocols, for the production and purification of 10s of milligram amounts of very pure, highly active and biophysically coherent untagged recombinant rat lactate dehydrogenase using the ÄKTAxpress™ liquid chromatography system. The automated 2-step protocol uses generic commercially available pre-packed columns and minimal buffers and has minimal user input. A novel protein crystallisation rationale was developed for fragments and small molecule.
